# Comparison of therapeutic effects of different acupuncture and moxibustion therapies on constipation after stroke treatment: A protocol for systematic review and network meta-analysis

**DOI:** 10.1097/MD.0000000000027397

**Published:** 2021-10-15

**Authors:** Jingwen Shang, Yongyang He, Rui Wang, Yifan Xu, Jia Xu

**Affiliations:** a Chengdu Eighth People's Hospital (Geriatric Hospital of Chengdu Medical College), Chengdu, Sichuan Province, China; b Tianjin University of Traditional Chinese Medicine, Tianjin, China.

**Keywords:** acupuncture and moxibustion, constipation, ischemic, network meta-analysis, protocol, stroke

## Abstract

**Background:**

Constipation is a common complication after stroke, which seriously affects patients’ quality of life and recovery. Many evidences show that acupuncture and moxibustion therapy has advantages in the treatment of constipation after stroke. But different types of acupuncture and moxibustion have different effects, and there is no research to prove which one is more effective.

**Methods:**

According to the search strategy, we will retrieve the randomized controlled studies of acupuncture and moxibustion in the treatment of constipation after stroke from China Knowledge Network, Wanfang, VIP, China Biomedical medicine, PubMed, Embase, Web of Science, and The Cochrane Library databases. The retrieval time was from the establishment of the database to July 2021. Studies will be screened according to inclusion and exclusion criteria, and the quality of the studies will be assessed using the Cochrane Risk Bias Assessment Tool. All data analyses will be performed using Revman 5.4, Gemtc 0.14.3, and Stata 14.0. Finally, we will evaluate the strength of evidence using the Grading of Recommendations Assessment, Development, and Evaluation method.

**Results:**

In this study, the efficacy of different acupuncture and moxibustion therapies in the treatment of constipation after stroke will be evaluated by evaluating defecation frequency, stool property score, constipation symptom score, quality of life score, adverse reactions, etc.

**Conclusions:**

This study will provide reliable evidence-based evidence for selecting the best acupuncture and moxibustion therapy for constipation after stroke.

## 1 Introduction

Stroke is one of the leading causes of death and disability worldwide,^[1]^ and approximately 22 million people worldwide experience stroke every year.^[2]^ Constipation, a common complication of stroke with an incidence of up to 48%, is an independently high-risk factor for ischemic stroke. The incidence of constipation in the recovery period is higher than that in the acute period, which also hinders the rehabilitation of patients and seriously affects their social functions and quality of life.^[3]^ Constipation can also cause negative effects on mental and physical health of patients, including low mood, limited social activities, lower quality of life, resulting in adverse outcomes and increasing medical costs, etc.^[4,5]^

The current treatment of post-stroke constipation is based on the treatment of functional constipation, including stool softeners, stimulants, permeable and stimulative laxatives, lifestyle changes, dietary structure adjustment, enemas, etc.^[6]^ However, the conventional treatment may produce adverse reactions including abdominal distension, dehydration, etc. And it is prone to relapse after drug withdrawal.^[7]^ According to a European survey on the satisfaction with the treatment plan for chronic constipation, most patients are not satisfied with the current therapeutic regimen and hope to seek other complementary or alternative plans.^[8]^

As an important part of alternative and complementary medicine, acupuncture is increasingly used in the treatment of constipation in western countries.^[4]^ A number of evidence-based studies have confirmed the reliability and safety of acupuncture in treating constipation.^[9,10]^ Modern studies have confirmed that stimulation of acupoints can regulate nerves and body fluids, promote intestinal peristalsis, and stimulate parasympathetic nerve, thus increasing rectal pressure and restoring the sense of defecation.^[7]^ However, there are also many forms of acupuncture and moxibustion therapies, such as needle acupuncture, electroacupuncture, moxibustion, etc. Although they are all based on the meridian theory of traditional Chinese medicine, their therapeutic effects vary with different treatment forms. For example, electroacupuncture is a therapy that adds electrical stimulation on the basis of traditional acupuncture, which combines the advantages of traditional acupuncture's meridian theory and low-frequency current.^[11]^ Clinical studies have confirmed that electroacupuncture has better efficacy than Western medicine in treating post-stroke constipation.^[12]^ Moxibustion achieves the therapeutic effect of stimulating acupoints through the thermal effect produced by moxa stick burning. Compared with western medicine, moxibustion can increase the number of defecation and reduce the score of stool symptoms.^[13]^ Existing evidence shows that different forms of acupuncture and moxibustion are superior to western medicine in the treatment of post-stroke constipation, but it is not possible to judge which form of acupuncture and moxibustion is better in the treatment of post-stroke constipation. Standard meta-analysis cannot solve this problem, while network meta-analysis (NMA), an advanced meta-analysis technology developed based on standard meta-analysis, can compare multiple competing interventions in a single statistical model, and compare and rank the advantages and disadvantages of multiple clinical interventions.^[14,15]^ Therefore, NMA will be used in this study to explore the difference in efficacy of different acupuncture and moxibustion therapies on post-stroke constipation, in order to provide evidence for the selection of optimal acupuncture and moxibustion treatment scheme for the clinical treatment of post-stroke constipation.

## 2 Methods

### 2.1 Protocol register

This NMA was conducted according to the Preferred Reporting Items for Systematic Reviews and Meta-Analyses for NMA guidelines.^[16]^ Moreover, it has been registered on open science framework (Registration number: DOI 10.17605/OSF.IO/DZNSG).

### 2.2 Ethics

Since the protocol does not require patient recruitment and collection of personal information, it does not require ethics committee approval.

### 2.3 Eligibility criteria

#### 2.3.1 Types of studies

Only randomized controlled studies will be included, regardless of blinding, publication status, region, but language will be restricted to Chinese and English.

#### 2.3.2 Types of participants

We will include adult patients (18 years and older) who are constipated after a first or recurrent stroke, without limiting stroke type (ischemia or hemorrhage) or stage (acute, subacute, or chronic). Acute and subacute stroke were defined within 6 months of onset, and chronic stroke was defined >6 months after onset.^[17]^ The diagnostic criteria for constipation refer to the description of constipation in Rome II–III criteria^[18,19]^ or Guidelines for Clinical Research on Chinese New Herbal Medicine.^[20]^

#### 2.3.3 Intervention measure

The treatment group was treated with different acupuncture and moxibustion treatment scheme, including conventional acupuncture, warm acupuncture, electroacupuncture, fire acupuncture, moxibustion, acupoint embedding, and acupoint injection. The control group was given western medicine or placebo.

#### 2.3.4 Outcome indicator

(1) Primary outcome indicator: defecation frequency, defined as the average number of spontaneous defecations per week.
(2) Secondary outcome indicators: Stool trait scores (such as Bristol Stool Form Scale), constipation symptoms scores, quality of life score (such as the patient assessment of constipation quality of life), adverse reactions (defined as treatment-related uncomfortable symptoms occurring during treatment).

### 2.4 Exclusion criteria

(1) Studies published repeatedly;
(2) Studies whose literatures are abstract, review, and animal research;
(3) Studies whose treatment group includes other Chinese medicine treatment programs (such as massage, cupping, etc);
(4) Studies which is unable to extract relevant data from published results, and unable to obtain original data after contacting the author;
(5) Studies in which intervention measures are inconsistent with the study.

### 2.5 Search strategy

The 2 researchers will search independently through search databases, including China Knowledge Network, Wanfang Data Knowledge Service Platform, VIP Information Chinese Journal Service Platform (VIP), and China Biomedical Database. The retrieval time is from the establishment of the database to July 2021. Chinese keywords: “stroke” (*zhong feng*), “apoplexy” (*cu zhong*), “constipation” (*bian mi*), “electroacupuncture” (*dian zhen*), “warm needle” (*wenzhen*), “fire needle” (*huozhen*), “moxibustion” (*aijiu*); English keywords: “constipation,” “astriction,” “electroacupuncture,” “electroacupuncture,” “warm needle,” “fire needle,” “moxibustion,” “stroke,” etc. The 2 researchers will select the included literature independently according to the inclusion and exclusion criteria. Any objections will be decided after consultation with the third researcher. PubMed retrieval strategies are shown in Table 1.

**Table 1:** Search strategy in PubMed database.

Number	Search terms
#1	Stroke [MeSH]
#2	Cerebrovascular Accident [Title/Abstract]
#3	Cerebrovascular Apoplexy [Title/Abstract]
#4	Brain Vascular Accident [Title/Abstract]
#5	Cerebrovascular Stroke [Title/Abstract]
#6	Apoplexy [Title/Abstract]
#7	Acute Cerebrovascular Accident [Title/Abstract]
#8	#1 OR #2 OR #3 OR#4 OR#5 OR #6 OR #7
#9	Constipation [MeSH]
#10	Dyschezia [Title/Abstract]
#11	Colonic Inertia [Title/Abstract]
#12	#9 OR #10 OR #11
#13	Acupuncture [MeSH]
#14	Acupuncture [Title/Abstract]
#15	Pharmacopuncture [Title/Abstract]
#16	Electro-acupuncture [Title/Abstract]
#17	Warm needle [Title/Abstract]
#18	Fire needle [Title/Abstract]
#19	Blood-letting puncture [Title/Abstract]
#20	Moxibustion [MeSH]
#21	Moxibustion [Title/Abstract]
#22	Acupoint catgut embedding [Title/Abstract]
#23	#13 OR #14 OR #15 OR #16 #17 OR #18 OR #19 OR #20 OR #21 OR #22
#24	#8 AND #12 AND #23

### 2.6 Data screening and extraction

**Figure 1:**
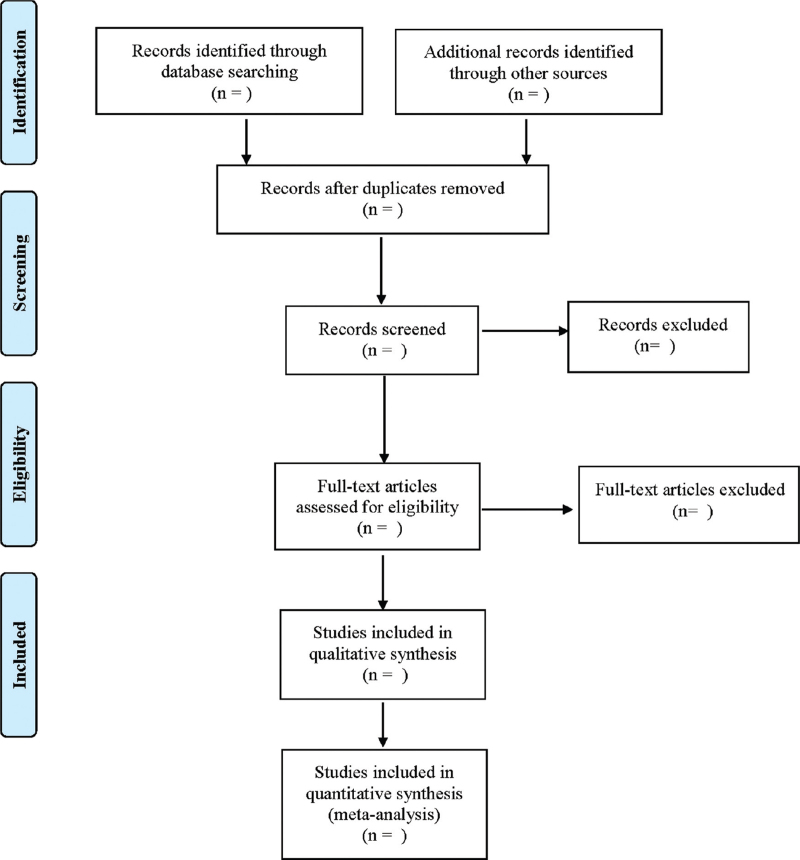
Flow diagram.

Two researchers will read titles and abstracts according to the above inclusion and exclusion criteria, and initially screened out the literature that might meet the criteria. After the removal of duplicate literature, further reading of the full text, independent evaluation of literature quality. Finally, inclusion will be determined. Any dispute shall be settled through consultation or with a third party. The data included author, year of publication, study type/level of evidence, sample size, sex, age, course of disease, interventions, course of treatment, and outcome indicators. The literature screening process is shown in Fig. 1.

### 2.7 Literature quality assessment

The Cochrane Collaboration risk bias assessment tool will be used to assess the risk bias of the included randomized controlled studies, including the following 7 aspects: random sequence generation, allocation concealment, participant blinding, outcome assessor blinding, incomplete outcome data, selective reporting, and other sources of bias. Each domain will be rated as having a high, low, or unclear risk of bias as appropriate.

### 2.8 Statistical analysis

#### 2.8.1 Data analysis and processing

The Stata14.0 software will be used to map the evidence network to show the comparison of outcomes interventions. We will use GeMTC14.3 based on The Bayesian framework for mesh meta-analysis. The effect value of dichotomous variables is represented by odd ratio (OR), and the effect value of continuous variables is represented by mean different (MD). The results of statistical analysis will be expressed with 95% confidence interval (CI). The Markov Chain Monte Carlo (MCMC) fitting consistency model will be used for Bayesian inference. Four chains will be used for simulation, and the number of iterations is set as 50,000 times (the first 20,000 times for annealing and the last 30,000 times for sampling). The potential scale reduction factor (PSRF) was used to reflect the convergence degree of the model. When PSRF was close to or equal to 1, it indicated that the data convergence was good and the obtained results were highly reliable.

#### 2.8.2 Assessment of inconsistency

When there was a closed ring between each intervention, the inconsistency test was conducted. Stata14.0 was used for Z test to evaluate the consistency of the results of direct comparison and indirect comparison. If *P *≥* *.05, the possibility of inconsistency between direct comparison and indirect comparison was small. If *P* < .05, there is a greater possibility of inconsistency between direct comparison and indirect comparison, and fitting inconsistency analysis is required. We used Stata14.0 to calculate the surface under the cumulative ranking curve of different interventions, and the larger the cumulative ranking curve value, the better the efficacy of the intervention. Finally, a comparative-correction plot will be drawn to assess whether the small sample effect exists.

#### 2.8.3 Dealing with missing data

If the data for the study is incomplete or not reported in the study, the researchers will contact the first or corresponding author of the study by telephone or email. If the required data cannot be obtained, descriptive analysis will be used instead of meta-analysis, and these studies will be excluded if necessary.

#### 2.8.4 Assessment of reporting biases

The comparison-adjusted funnel plots were obtained with the specific ranking order to detect small sample size study effects and publication bias. All analyses were conducted using R V.3.6.1 with the GeMTC package.

#### 2.8.5 Evidence quality evaluation

We will use the Grading of Recommendation Assessment, Development and Evaluation scoring method to grade the evidence of the outcome indicator.^[21]^ Based on randomized controlled trials, a quality rating of “high” is the highest level of evidence. The evaluation content includes bias risk, indirectness, inconsistency, inaccuracy, and publication bias. And the quality of evidence will be rated as high, medium, low, or very low.

## 3 Discussion

At present, the pathophysiology process of constipation in stroke patients is not completely clear, and it is believed to be closely related to factors such as impaired autonomic nervous function, changes in defecation environment, changes in eating patterns and types, decreased activity, complications, drugs, etc.^[5,22]^ Related studies have shown that constipation after stroke can affect the removal of metabolic waste, increase the risk of cerebral vasospasm and nerve injury, and induce the recurrence of cardiovascular and cerebrovascular diseases.^[23,24]^

Acupuncture and moxibustion therapies have been used in the treatment of constipation for a long time, and clinical studies have found that long-term use of acupuncture and moxibustion can help the gastrointestinal function recovery of stroke patients, with a two-way regulatory effect.^[25]^ The impaired autonomic nervous function of stroke patients can lead to abnormal defecation reflex or insufficient autonomic defecation motivation, resulting in constipation. Acupuncture can regulate the signal transduction of the autonomic nervous system, bidirectional regulation of gastrointestinal digestion, and absorption function and intestinal flora distribution, so as to improve constipation symptoms.^[26]^ Current studies mainly focus on the comparison of efficacy between acupuncture and moxibustion and other treatment schemes, but lack of efficacy comparison between acupuncture and moxibustion. NMA can produce “indirect evidence” for a potential comparison where a head-to-head comparison is unavailable.^[27]^ Moreover, effect estimates from Network meta-analyses can be linked to probabilistic models, allowing ranking based on which treatments are most effective for outcomes of interest.^[15]^ This is an effective solution to our problem.

However, there are some limitations in our study: due to the limitation of language retrieval, we will only include Chinese and English literature, which may ignore studies in other languages and regions; different factors such as stroke stage, acupoint and course of treatment may increase the possibility of heterogeneity. Nevertheless, we believe that the results of this study will help to find the best acupuncture and moxibustion treatment for post-stroke constipation.

## Author contributions

**Data collection:** Jingwen Shang and Yongyang He.

**Funding support:** Jia Xu.

**Literature retrieval:** Jingwen Shang and Yongyang He.

**Software operating:** Rui Wang, Yifan Xu.

**Supervision:** Jia Xu.

**Writing – original draft:** Jingwen Shang, Yongyang He.

**Writing – review & editing:** Jingwen Shang, Jia Xu.

## References

[1] Strong K Mathers C Bonita R. Preventing stroke: saving lives around the world. Lancet Neurol 2007;6:182–7. 17239805 10.1016/S1474-4422(07)70031-5

[2] Song S Liang L Fonarow GC. Comparison of clinical care and in-hospital outcomes of Asian American and white patients with acute ischemic stroke. JAMA Neurol 2019;76:430–9. 30667466 10.1001/jamaneurol.2018.4410 PMC6459126

[3] Li J Yuan M Liu Y. Incidence of constipation in stroke patients: a systematic review and meta-analysis. Medicine (Baltimore) 2017;96:e7225. 28640117 10.1097/MD.0000000000007225 PMC5484225

[4] Zhai J Mu W Si J. Acupuncture for constipation in patients with stroke: protocol of a systematic review and meta-analysis. BMJ Open 2018;8:e020400. 10.1136/bmjopen-2017-020400 PMC5884333 29602854

[5] Su Y Zhang X Zeng J. New-onset constipation at acute stage after first stroke: incidence, risk factors, and impact on the stroke outcome. Stroke 2009;40:1304–9. 19228840 10.1161/STROKEAHA.108.534776

[6] Zhou SL Zhang XL Wang JH. Comparison of electroacupuncture and medical treatment for functional constipation: a systematic review and meta-analysis. Acupunct Med 2017;35:324–31. 28630049 10.1136/acupmed-2016-011127

[7] Zhang C Guo L Guo X. Short and long-term efficacy of combining Fuzhengliqi mixture with acupuncture in treatment of functional constipation. J Tradit Chin Med 2013;33:51–9. 23596812 10.1016/s0254-6272(13)60100-4

[8] Müller-Lissner S Tack J Feng Y. Levels of satisfaction with current chronic constipation treatment options in Europe - an internet survey. Aliment Pharmacol Ther 2013;37:137–45. 23126338 10.1111/apt.12124

[9] Wang L Xu M Zheng Q. The effectiveness of acupuncture in management of functional constipation: a systematic review and meta-analysis. Evid Based Complement Altern Med 2020;2020:6137450. 10.1155/2020/6137450 PMC7317618 32655664

[10] Zhu L Ma Y Deng X. Comparison of acupuncture and other drugs for chronic constipation: a network meta-analysis. PLoS One 2018;13:e0196128. 29694378 10.1371/journal.pone.0196128 PMC5918910

[11] Comachio J Oliveira Magalhães M Nogueira Burke T. Efficacy of acupuncture and electroacupuncture in patients with nonspecific low back pain: study protocol for a randomized controlled trial. Trials 2015;16:469. 26472590 10.1186/s13063-015-0850-7 PMC4608106

[12] Liu WA Wu QM Li XR. Therapeutic effect of electroacupuncture on constipation after apoplexy. J Clin Acupunct Moxibust 2008;17–8.

[13] Wang M Ren H Zhu J. Effect analysis of moxibustion combined with rhubarb application on shenque point for post-stroke constipation. J New Chin Med 2017;49:118–20.

[14] Caldwell DM Ades AE Higgins JP. Simultaneous comparison of multiple treatments: combining direct and indirect evidence. BMJ 2005;331:897–900. 16223826 10.1136/bmj.331.7521.897 PMC1255806

[15] Lu G Welton NJ Higgins JP White IR Ades AE. Linear inference for mixed treatment comparison meta-analysis: a two-stage approach. Res Synth Methods 2011;2:43–60. 26061599 10.1002/jrsm.34

[16] Hutton B Salanti G Caldwell DM. The PRISMA extension statement for reporting of systematic reviews incorporating network meta-analyses of health care interventions: checklist and explanations. Ann Intern Med 2015;162:777–84. 26030634 10.7326/M14-2385

[17] Momosaki R Yamada N Ota E Abo M. Repetitive peripheral magnetic stimulation for activities of daily living and functional ability in people after stroke. Cochrane Database Syst Rev 2017;6:CD011968. 28644548 10.1002/14651858.CD011968.pub2 PMC6481821

[18] Mearin F Lacy B Chang L. Bowel disorders. Gastroenterology 2016;5:5085. 10.1053/j.gastro.2016.02.031 27144627

[19] Longstreth GF Thompson WG Chey WD. Functional bowel disorders. Gastroenterology 2006;130:1480–91. 16678561 10.1053/j.gastro.2005.11.061

[20] Zheng XY. Guiding Principles for Clinical Research of New Chinese Medicine. 2002;Beijing: China Medical Science Press, 378–398.

[21] Puhan MA Schünemann HJ Murad MH. A GRADE Working Group approach for rating the quality of treatment effect estimates from network meta-analysis. BMJ 2014;349:g5630. 25252733 10.1136/bmj.g5630

[22] Yang Y Zhong DQ Shen YY. Analysis of factors influencing defecation in patients with acute stroke. Chin Nurs Res 2018;32:3870–4.

[23] Bracci F Badiali D Pezzotti P. Chronic constipation in hemiplegic patients. World J Gastroenterol 2007;13:3967–72. 17663511 10.3748/wjg.v13.i29.3967 PMC4171169

[24] Zhang T Wang G Li B. Effect of acupuncture for constipation after ischemic stroke: study protocol for a randomized controlled trial. Trials 2018;19:454. 30134942 10.1186/s13063-018-2750-0 PMC6106942

[25] He SM Song R Su TS. Explore the mechanism and treatment analysis of acupuncture and Moxibustion for constipation after stroke. J TMR Clin Res 2021;4:03.

[26] Wang L-l. Observation on therapeutic effect of acupuncture and moxibustion on constipation after ischemic apoplexy. Guide China Med 2020;18:185–6.

[27] Salanti G Higgins JP Ades AE Ioannidis JP. Evaluation of networks of randomized trials. Stat Methods Med Res 2008;17:279–301. 17925316 10.1177/0962280207080643

